# 

**Published:** 1890-10-25

**Authors:** 


### NOTICE TO CORRESPONDENTS.

All MS., Letters, Books for Review, and other matters intended for the Editor should be addressed The Editor, The Lodge, Porchester Square, London, W.

The Editor cannot undertake to return rejected M.S., even when accompanied by stamped directed envelope.

### Notice to Advertisers and Subscribers.

All Advertisements, Orders for Copies of The Hospital, and Business Communications should be addressed to The Publisher, *not to the Editor*, The Hospital, 140, Strand, W.C.

### Bound Volumes of "The Hospital."

Vol. VI., containing full page portraits of T.R.H. the Prince and Princess of Wales, and Miss Florence Nightingale, is still obtainable.

Vol. VII. and all the earlier volumes are also for sale.

Orders will be received for Vol. VIII., 4s. post free. Cases for binding may be had of the Publisher, at 1s. 6d. each.

### To Residents, Secretaries, and Matrons.

The Editor will be glad to have the Regulations and each Report as issued by Asylums, Hospitals, Convalescent and Nursing Institutions, as well as those of other Charities, and an early copy in duplicate of all Appeals and Festival Notices, etc., addressed to *The Editor, The Lodge, Porchester Square, London, W.* Any superintendent, secretary, matron, or other chief official who regularly sends such information may be registered, on application to the Publisher, to receive free of cost a cover for binding The Hospital in half-yearly volumes.

### BURDETT'S HOSPITAL ANNUAL FOR 1890

May be obtained of the Publisher, The Hospital Offices, 140, Strand, W.C., price 2s. 6d. limp boards, or 3s. 6d. cloth. All orders must be accompanied by a remittance.

### General Charities.

[This Column is devoted to an account of work of charitable institutions other than Medical Charities. The Editor will be glad to receive reports or news of work at 140, Strand. *Philanthropy* should be plainly written on the envelope.]

Mrs. Evans, who for fifteen years has been matron of the **MIDDLEMORE EMIGRATION HOMES** has had a cheque for eighty pounds presented to her on leaving.

**THE CHURCH OF ENGLAND TEMPERANCE SOCIETY** has in the press, and will shortly publish under the title of "The Nations' Hope," a practical handbook for band of hope workers and others having control of the education of the young. The papers have been written by authors who in each department have had practical experience of their subject. It specially deals with the interesting adjuncts to usual Band of hope work, such as gymnastics, musical drill, boys' brigade, ambulance training, industrial work, exhibitions, &c. The work, which will supply a long-felt want, will be fully illustrated, and published at a price within the reach of all.

Out of the twelve or thirteen thousand men licensed as cab drivers in London, about 8,000 are actually employed. Of these 1,200 are members of the **CAB DRIVERS' BENEVOLENT ASSOCIATION**, and contribute a yearly subscription of five shillings There are now 51 annuitants on the books of the association, each in receipt of £20 per annum, who, but for this help, would have been reduced to refuge in the workhouse. It is no pauperising, this scheme of the association, a man must contribute his subscription for five years before he can become a candidate for an annuity, and the committee are issuing a strong appeal to the public just now to help increase this annuity fund, and help these servants who may growl a bit at a close fare sometimes, but who are in valuable to our comfort, and martyrs in their old age to lung disease and rheumatism. Hon. Sec., G. Stormont Murphy, Esq., 15, Soho Square, W.

**THE SOCIETY OF SCHOOLMASTERS**, which was originally formed nearly one hundred years ago on a principle of life assurance, for the benefit of such subscribing members as were masters of endowed or boarding schools, combined with a charitable fund for the relief of distressed subscribers, their widows, and orphans, was converted into a purely charitable institution so long ago as 1821. Its benefits are now extended to necessitous masters of all schools, not coming under the Elementary Education Act, their widows and orphans, provided that the masters have been continuously engaged in teaching for not less than five years. Priority of consideration is given to the application of subscribers. The qualification of membership was, till within the past five or six years, a donation of five guineas paid in one sum, or an annual subscription of one guinea. Owing to the fact that for so many years past the subscriptions and donations have remained stationary, or nearly so, the annual subscription was reduced to half a-guinea, and the benefits of the society were more widely extended in the hope of inducing more of the profession to support its work. But the result has not justified the expectations of the managing committee, They looked for a spirit of self-help in a craft which apparently does not possess it in the quantity it should, that is, judging by the small list of subscribers, which does not contain a tithe of the names of even the masters of the principal grammar schools in the kingdom. A good example has been set by the masters of Clifton College, who, during the past five years, have sent through their Secretary a lump subscription. This might with advantage be followed by many other schools, especially the smaller ones, where the pay of the staff is not high. Many a man, we believe, who would find even an annual subscription of half-a-guinea a tax on his slender means, would gladly give a few shillings towards a subscription from the staff of which he is a member. If the society were to meet with due support from the profession its income would be, as it should be, treble what it now is. The need of more generous support is amply testified by the fact that the average number of cases relieved has during the past six years been exactly doubled. The expenses of disbursement are remarkably small, and the society has not adopted the objectionable system of voting for the decision of the merits or claims of applicants, which are considered by a committee of schoolmasters, who hold a high position in their profession. Many of the cases which come under their notice are of a very distressing nature; paralysis and blindness only too often overtake members of the craft, whose income is not such, in the majority of cases, as to allow of any savings. Not only is temporary aid given in order to assist in tiding over some passing trouble, but pensions of varying amounts are granted. These latter seldom exceed £25 in value; in fact, by the rules of the society no grant shall, under any circumstances, exceed £50. We notice that there is no limit set to the age at which pensions may be granted; whether this is advisable from a financial point of view with a society possessing so small an income on which there are so many claims, is, we think, open to dispute, and from another point of view still more so. Self-reliance seems to be one of those virtues which is most easily sapped, and anything which tends in the slightest degree to pauperise should be avoided. But the society does some excellent work, and is worthy of more support. Donations can be sent to the Treasurer, the Rev. Dr. Baker, Merchant Taylors' School, Charterhouse Square, E.C.

### Scraps and Gleanings.

North Cheam is suffering from an epidemic of diphtheria.

Miss Caine, daughter of Mr. Caine, M.P., is studying medicine.

A life of Laura Bridgeman, the celebrated deaf-mute, is in the press.

The annual ball in aid of the Royal Free Hospital will be held on December 2nd.

The Quarantine Board declares that Mecca, Medina, Jeddah, and Yambo are now free from cholera.

The committee of the Society for the Prevention of Cruelty to Children are sending out special written letters appealing for funds.

Lord Derby presided at the annual meeting of the Liverpool centre of the St. John Ambulance Association, and gave an interesting address.

A concert in aid of the North West London Hospital was given last week, chiefly through the aid of Mr. Harry Bewsher and Mr. Edmund Barnes.

Norwood Cottage Hospital reports 125 patients treated during the last twelve months. A bed has been endowed to the memory of the late Emperor Frederick.

It was resolved at a meeting of the medical profession of Portsmouth to invite the British Medical Association to hold its annual conference at Southsea in 1891.

Bristol Medical School will hold its annual dinner at the Montague Hotel, Kingsdown, on November 1st, at seven p.m., Mr. F. Richardson Cross, F.R.C.S., in the chair.

Four ladies have just been admitted to the list of doctors of the Paris faculty of medicine. One of them is of Polish nationality, and intends to practise at Warsaw.

The Portsea bazaar in aid of the hospital has been an enormous success; the institution will benefit to the extent of £2,000. All concerned are to be congratulated.

Mr. William Ernest Marshall has been appointed Junior House Surgeon to the Royal Portsmouth, Portsea, and Gosport Hospital, vice Mr. Edward Cooper, appointed Senior House Surgeon.

At Cambridge University, Mr. L. R. Wilberforce, M.A., of Trinity College, has been appointed demonstrator of experimental physics, and Mr. E. Lloyd Jones, M.A., of Downing College, demonstrator in pathology.

The quarterly board of Oldham Infirmary had a good report to receive: the expenses of the Hospital Saturday collection had been less and the receipts greater. Over 500 accidents were treated during the quarter.

The Duchess of Fife has signified her acceptance of the office of president of the Edinburgh School of Medicine for Women, which is the first school where a medical education has been afforded to women in Scotland.

The Guild of St. Luke held its festival last Friday night at St. Paul's Cathedral, when Canon Newbolt preached an impressive sermon. The Guild now numbers 400 members, who believe that the art of healing includes that of both body and soul.

Royal Hospital for Incurables.—If any of our readers have votes for the November election for a pension of £20, we beg to call their attention to the case of Sarah Ward, which is strongly recommended by one of the Devonshire Square nursing sisters.

Dr. Richardson was entertained to breakfast on the 14th inst. at the Royal College of Physicians in Kildare Street, Dublin. Sir Wilfrid Lawson and other supporters of temperance, Mr. Croly, president of the Royal College of Surgeons, and many others were present.

Leicester Infirmary held its quarterly court on the 14th inst. Mr. Johnson called attention to the fact that they had fourteen cases of typhoid fever from Oadby; each case cost £12 or £14. He hoped the people of Oadby would remember this on Hospital Sunday.

Miss C. H. Graham, M.D., and Miss Anna Baumler, M.D., former students of the London School of Medicine for Women, have been appointed lady doctors under the Countess of Dufferin's Fund in India. The former will serve at Rangoon, and the latter at Lahore.

On Saturday afternoon the Baroness Burdett Coutts and Mrs. W. H. Collingridge laid the memorial stones of the extension wing of the Printers' Almshouses at Wood Green, in the presence of a numerous assembly. Amongst those present were Sir Polydore De Keyser, Mr. Burdett Coutts, M.P., and Mr. W. H. Collingridge.

Hulme possesses a Working Men's Sanitary Association, which might serve as a model to other towns. Lectures and meetings are held through out the winter. Complaints are investigated by the honorary officers of the association, and, when necessary, notified to the City authorities. The hon. secretary is Mr. John Lotherington, 202, Chester Road.

The Hungarian Chamber has introduced a Bill which provides that workmen, in case of illness, would be entitled to gratuitous medical attendance, and even to assistance in money during 20 weeks. The funds would be maintained by a charge of 2 or 3 per cent. on the wages of the workmen, for the due retention of which the employers would be responsible. No assistance would be forthcoming in cases of illness caused by drunkenness or debauchery or in any intentional manner.

Dr. Conry, of the Salford Union, has been presented by the infirmary staff with two bronze equestrian statues and a handsome marble clock, the latter bearing the following inscription: "Presented to Dr. J. T. C. Conry, First Medical Superintendent of the Salford Union Infirmary, by the officers and nursing staff, in recognition of his never-failing kindness to the donors, and of eight years' conscientious and untiring discharge of duty to the patients" Dr. Conry is reported to have said in reply: "I thank you all heartily for the handsome present, and I take this opportunity of expressing my appreciation to the staff for the continued obedience, attention, and respect it has shown me during a period when my good name was attacked and my authority in the hospital undermined. It is very gratifying to receive such evidence of good will towards myself, and to feel, notwithstanding the disparaging comments on the nursing and management of this institution, we are conscious that our duties have been honestly and effectively performed."

### The Medical Students' Supplement.

[All communications for this Supplement should be addressed to The Editor of The Hospital, at 140, Strand, with the words "Students' Column" written in the left hand top corner of the envelope.]

### IN THE CAUSE OF MEDICAL STUDENTS.

The Editor of The Hospital has long had a desire to devote some columns of the paper to the interests of medical students, but there have always been difficulties in the way. The demands for The Hospital have, however, so much increased, that it has been determined to give up at least four columns each week entirely for the use of the students. In order to effect this, it has become necessary to increase and reorganise the pages of the paper. In the first place, information that is intended for people interested in hospitals and their working, apart from medical practitioners, will be transferred from November 1st to the Nursing Mirror supplement, which will be increased in size. By this arrangement it will be possible for The Hospital proper to be mainly devoted to medical and scientific topics without sacrificing any of the special features which have caused the paper to attain so wide a popularity.

In the Students' Supplement the Editor will endeavour to report from week to week the meetings of the various students' medical societies held at the different hospitals. He hopes that the secretaries of these societies will themselves send him regularly the minutes of their meetings, an abstract of the papers read, together with some notes on the discussion following the papers. It often happens that some very interesting papers are read at the students' medical societies which have hitherto, with the exception of one or two hospitals which print the proceedings of their societies themselves, been lost, for want of publication.

The Editor would also be pleased to receive the results of football matches and other athletic sports and games from the secretaries of the various clubs, together with any other Hospital news interesting to students generally.

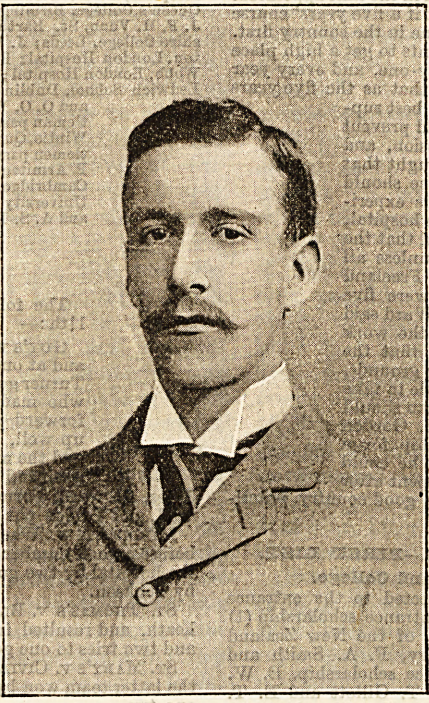

Mr. Frederick E. Hopkins (Guy's).
Exhibitioner and Gold Medallist in Inter. M.B. London University.

### NOTES FROM THE SCHOOLS.

### EDINBURGH UNIVERSITY.

The School of Medicine was opened on Tuesday, October 14th. Several of the professors gave the first lecture of their courses.

Professor Grainger Stewart told the students that they had to learn how to fight death and disease, and showed a series of tables illustrating the proportion of the death-rate to the density of the population, age, and sex in different countries. In relation to the mortality due to the various diseases, he pointed out how tubercular diseases were the worst enemies. Eight years ago he announced Koch's discovery of the bacillus of consumption, and now he had to tell them that, at the recent Berlin Congress, Koch had announced that he had discovered a substance which would not only prevent the growth of the tubercular germ, but also kill it when it had once started.

Professor Chiene, in his lecture, dwelt on the importance of the practical and tutorial classes in surgery. No student could learn to apply a bandage by simply watching someone else put it on. He told the junior students that their second year was the time to learn surgery; in the first year they had the first professional, and in the third year the second professional, and in the fourth year they had medicine to study.

Professor Rutherford took as the subject of his address in physiology "The Attainment of the Physiological Ideal." He said we must all try to improve ourselves by proper training; but although hereditary influence had much to do with the attainment of a physiological ideal, it would not do for a man with an illustrious father to suppose that there was no need for him to exert himself, any more than for a man with non-illustrious parents to expect that he could do nothing. Students who wished to succeed must live carefully, take proper exercise, and exercise their brains well, but not too much.

Professor Greenfield compared the study of pathology to that of a foreign language, and then referred to the importance of the leucocytes which were said to be the builders of our bodies, to act as scavengers and policemen, and as soldiers to fight the microbes.

Professor Simpson presented the students with a copy of his graduation address delivered last August, and strongly urged them with the importance of a thorough knowledge of the diseases of women and children in daily practice.

Professor Sir William Turner referred to the scope of anatomy and its importance to the surgeon and to the physician. Professor Crum Brown, before proceeding to his lecture, referred in appropriate terms to the late Professor Sellar, and Professor Fraser proceeded to define the scope of materia medica without giving any introductory remarks.

### MEN OF THE FUTURE.

Mr. Frederick E. Hopkins, whose portrait we publish to-day, was engaged at work in the Toxicological Laboratory at Guy's Hospital before commencing medicine. He entered Guy's as a medical student in October, 1888, and did all the work for the London University examinations at that school. During the greater part of his two years at the hospital he has been connected with the editorial work of the *Guy's Hospital Gazette*. At the recent Intermediate Examination for the degree of M.B. at the London University, Mr. Hopkins obtained the Exhibition and Gold Medal in Chemistry, together with honours in Materia Medica and Pharmaceutical Chemistry. Such honours in the preliminary examination should point forward to the same or even greater ones in the final.

### STUDENTS' MEDICAL SOCIETIES.

### I.—Westminster Hospital Guthrie Society.

President—Mr. C. Stonham. Hon. Secretary—Mr. H. A. Stonham.

The opening meeting of this society was held on Thursday, October 9th, the president (Mr. C. Stonham) in the chair. Mr. Stonham having referred to the work of the society in the past, and to the satisfactory state of the funds, thanked the members for the honour they had done him in electing him as their president. He then showed a child who was attending in the out-patient department of the hospital, and had persistent fourth branchial clefts, the right being larger than the left. In this case there did not appear to be a free communication into the pharynx, as a probe could not be readily passed into the air passage. The proceedings terminated with a smoking concert, Messrs. Pearson, Mills, Evans, and Fleming being loudly applauded for their songs.

### II.—Middlesex Hospital Medical Society.

The opening meeting of this Society was held on October 16th, the president, Mr. Pearce Gould, in the chair. Mr. A. Lawson opened a debate entitled "That the proposed five years'

curriculum of medical study will be preferable to the present plan." In this he considered that four years was not enough. Firstly, because so much time was spent over anatomy; secondly, the waste of time for the holidays; this, and taking off twelve months for out and in-patient dressing, left only fifteen months to get up general and three for special subjects. He thought that the average man could not tell an upper from a lower pair of tooth forceps, and considered that men should have a scientific as well as a practical training. Patients usually think that the G. P. is not able to treat them when they have a serious complaint. Money was, of course, an object with many men. He was quite certain that the advantage of a longer course was that it afforded a greater certainty for diagnosis. Mr. Charles then spoke on the other side of the question. He considered that men who really worked hard could easily get through the work in the time. Those who fly high in the profession certainly ought to have a longer course. He thought that men should get away from the hospital as soon as possible before getting into a groove, and likened the G.P. and consultant to a series of circles one of which swallowed and cut into the other. If a man could use the fifth year in residence in the hospital it would be well enough, but in cases where these appointments are only open to qualified men this was impossible. Mr. Seed said that there were always some lazy men in every school who could never get through the work in the time appointed. Mr. King, who spoke next, considered that if a five years' course were instituted, one of these should be done in the country first. Mr. G. Malby mentioned that if a man wants to get a high place in the services he had to get in at twenty-one, and every year made a difference. Mr. Thomas thought that as the five years could not affect them personally they had best support it; that the extra year's expense would prevent too many men from entering the profession, and this would be good; but he certainly thought that if five years were compulsory another degree should be given. Mr. E. Madge then related his experiences: he had already been four years at the hospital, and had not passed. Mr. Pitter considered that the five years would drive more men north, unless all licensing bodies adopted the plan. Mr. Freeland thought that now the majority of men were five years in getting through the work. Mr. Ward said it was too much of a rush to get through the work in the time appointed. Mr. Fardon said that the extension must be made purely on public grounds, namely, that the ordinary man was unable to take care of a case. He would suggest that a man should have another year after qualifying. Mr. Gordon Brodie thought it would be a great hardship for a man to have to spend a year more before he could earn his bread, though no doubt a year spent after qualifying, either in a hospital or with a good country practitioner, would be a great advantage.

### SCHOLARSHIPS AND PRIZES.—FIRST LIST.

### St. Bartholomew's Hospital and College.

The following gentlemen have been elected to the entrance scholarships and exhibitions: The senior entrance scholarship (1) in chemistry and physics, Sinclair Gillies, of the New Zealand University; (2) in biology and physiology, F. A. Smith and Horton Smith, equal. The junior entrance scholarship, E. W. Groves, University College, Bristol; H. T. Gillett and E. T. Toye, proximé accesserunt. Preliminary scientific exhibition, M. G. Pearson, University College School and St. Bartholomew's Hospital. Jeaffreson exhibition, G. Calvert, King's College School, London.

### Charing Cross Hospital Medical School.

The first entrance scholarship of 100 guineas has been awarded to M. Molley, and the second of 50 guineas to J. R. Langley.

### Middlesex Hospital Medical School.

The names of the successful candidates in the competition for the entrance scholarships are: First, A. E. Walter, £100; second, H. P. Noble, £60.

### Westminster Hospital.

The winter entrance scholarships have been awarded to A. T. White, £80, and A. J. Smith and A. H. Evans, £40, equal.

### London School of Medicine for Women.

Entrance scholarship, Miss Charlotte Hull, £30, undergraduate of the Royal University of Ireland. Miss Hull has recently returned from Kashmir, having worked under the late Dr. Fanny Butler, of Trinaghur. The Stuart Mill scholarship, Miss Ethel Bentham, £30 for four years, open only for students preparing to practise medicine in India under the Countess of Dufferin's Fund.

### King's College, London.

The Sambrooke science exhibitions of £60 and £40 have been awarded to Mr. A. S. McSorley and Mr. F. H. Jacob; the Clothworkers' science scholarships to P. Turner £100, and G. H. Landsdowne £50. The Warneford scholarships in general literature of £50 each to J. M. Scaub, F. C. M. Clifford, M. M. Townsend, M. B. Bell, and H. A. Good.

### PASS LISTS.

### Royal Colleges of Physicians and Surgeons.

The following gentlemen have passed the first professional examination of the Board in elementary anatomy and elementary physiology, viz.:—

Messrs. T. H. Agnew, student of University College, Liverpool; H. R. Andrews, London Hospital; H. Ashton, Glasgow University; F. W. Bailey, University College, Liverpool; G. R. Baldwin, St. George's Hospital; R. W. Bentley, St. Bartholomew's Hospital; J. R. Bishop, Owens College, Manchester; N. W. Brook, University College, Liverpool; A. D. Cockerton, London Hospital; F. W. E. Courtenay, Owens College, Manchester; R. A. Cowie, Cambridge University; J. S. Crabbe and Sydney Crompton, Queen's College, Birmingham; J. L. F. de Gannes, University College; H. G. Ellery, Queen's College, Cork; R. D. Evans, St. Mary's Hospital; R. H. Fagge, Guy's Hospital; R. W. Gilmour, St. Bartholomew's Hospital; J. Gorman, Royal College of Surgeons, Dublin; W. Grummitt, Guy's Hospital; S. C. Hague, Owens College, Manchester; M. L. G. Hallwright, and A. E. Hancock, Queen's College, Birmingham; C. E. Hibbard, Guy's Hospital; C. Homer, St. Mary's Hospital; F. Hughes, Queen's College, Birmingham; F. H. Humphris, Edinburgh University; C. D. Ingle, Bristol Medical School; J. H. Jolley, London Hospital; H. F. Joughin, Owens College, Manchester; G. B. Kaufmann, London Hospital; E. T. Lanyon, King's College; C. H. Maskew, Queen's College, Birmingham; E. G. Moon, St. Mary's Hospital; G. P. Parry, Charing Cross Hospital; L. S. Partridge, Oxford University; G. S. Pownall, St. Bartholomew's Hospital; G N. O. Slater, Sheffield Medical School; W. S. Snell, St. Mary's Hospital; S. B. Stedman, St. Thomas's Hospital; A. J. Thomson, Queen's College, Birmingham; P. Twomey, Owens College, Manchester; J. F. H. Venn, St. Bartholomew's Hospital; T. H. Waddington, Yorkshire College, Leeds; J. Wallace, Queen's College, Belfast; F. W. Walton, London Hospital; F. W. J. Webb, Charing Cross Hospital; W. S. Webb, London Hospital; L. T. Wells, St. Mary's Hospital; A. S. White, Ledwich School, Dublin; P. Wilkinson, Owens College, Manchester; and O. O. Williams, London Hospital. The following gentleman passed in elementary anatomy only, viz.: H. R. W. Wintle, Queen's College, Birmingham. The following gentlemen passed in elementary physiology only, viz: Messrs. F. Armitage, St. Bartholomew's Hospital; H. S. Basden, Cambridge and London Hospital; W. W. Hoare, Edinburgh University; J. T. Newton, Queen's College, Birmingham; and A. S. Turner, Guy's Hospital.

### ATHLETICS.

### Football.

The following matches were played on October 11th:—

Guy's v. Croydon, at Croydon.—Guy's kicked off, and at once invaded the Croydon lines, but Howard Turner got possession of the ball and passed to Flint, who made a good run to centre. After some good forward play, Guy's touched down. Guy's played up well, but were out-classed by their opponents, and the match ended in a victory for Croydon by a goal and a free kick by Marke and two tries to nil. Tries, Howard Turner and Flint. For Guy's, Cooper played well, and Briggs put in some fine work.

Middlesex v. Wasps.—Played at the Half Moon, Putney, before a good number of spectators, and ended in a victory for the hospital by two goals to nil. Tries by Marshall, goals kicked by Cookson.

St. Thomas's v. Blackheath.—At the Rectory Field, Blackheath, and resulted in a victory for the home side by one goal and two tries to one goal.

St. Mary's v. Civil Service.—Played at Wormwood Scrubbs; the latter team won by two goals and three tries to one goal and one try.

St. Bartholomew's v. Royal Military College.—At Sandhurst, the home team won easily by five goals and two tries to nil.

Dental Hospital v. Eltham House.—The latter won by one goal and two tries to nil.

University College Hospital (first) drew with Wasps (second) at Park. No score.

Guy's v. Vampires (a).—The hospital were beaten by two goals to nil.

St. Thomas's v. W. Kent.—The latter won by three goals to one.

Charing Cross v. Clapham Rovers (a).—The latter won by eight goals to nil.

St. Bartholomew's v. Old St. Stevens.—At Walthamstow, the latter won by four goals to one.

St. Bartholomew's v. Reigate Priory.—At Reigate, the Hospital were beaten by three goals to nil.

### EVENTS FOR THE MONTH OF OCTOBER.

[Items intended for this column must reach the office, 140, Strand, W.C., not later than first post on Monday mornings.]

### Medical Societies.

St. Bartholomew's Hospital, Abernethian Society.—October 30, Mr. D'Arcy Power, F.R.C.S., "The Cure of the King's Evil."

### Athletics.

Football Matches.—St. Thomas' Hospital first fifteen: October 29, v. Cambridge, at Cambridge. St. Thomas' Hospital second fifteen: October 25, v. Crusaders, at Ealing; King's College, Association: October 25, v. Old Salways, at Leyton; Rugby: October 25, v. Hampstead, at Wormwood Scrubbs.

